# The acute impact of a hematopoietic allograft on lung function and inflammation: a prospective observational study

**DOI:** 10.1186/1471-2466-13-2

**Published:** 2013-01-11

**Authors:** Alexandra Enocson, Richard Hubbard, Tricia McKeever, Nigel Russell, Jennifer Byrne, Emma Das-Gupta, Lynne Watson, Andrew W Fogarty

**Affiliations:** 1 Nottingham Biomedical Research Unit, Division of Epidemiology and Public Health, University of Nottingham, Clinical Sciences Building City Hospital, Nottingham NG5 1PB, UK; 2 Bone Marrow Transplant Unit, Department of Haematology, Nottingham University Hospital (City Campus), Nottingham, NG5 1PB, UK

**Keywords:** Lung function, Inflammation, Haematopoietic transplant

## Abstract

**Background:**

No studies have investigated the immediate impact of receiving an allogeneic hematopoietic stem cell transplant (HSCT) on pulmonary inflammation or lung function.

**Methods:**

Using a prospective study design, we quantified the changes in these outcome measures in eligible adult individuals in the first six months after receiving an allogeneic hematopoietic stem cell transplant.

**Results:**

Between January 2007 and December 2008, 72 patients were eligible to participate in the cohort, and of these 68 (94%) were included in the study. Compared to baseline, pulmonary inflammation as measured by exhaled nitric oxide increased after receiving a HSCT with the largest increment seen at three months (+6.0ppb, 95%CI: +0.4 to +11.5), and this was sustained at six months. Percent predicted forced expiratory volume in one second decreased over the same period, with the largest decrease observed at six weeks (−5.9%, 95% CI: -8.9 to −2.9), and this was also sustained over a six month period. Similar associations were observed for FVC. A larger increase in exhaled nitric oxide from baseline at six weeks and three months may be associated with decreased mortality (p=0.06, p=0.04 respectively).

**Conclusion:**

Our data demonstrate that recipients of an allogeneic HSCT experience an increase in biomarkers of pulmonary inflammation and a decrease in lung function in the first six months after the procedure. If independently validated in other study populations, these observations could have potential as a prognostic biomarker for this patient group.

## Background

The use of allogeneic hematopoietic stem cell transplant (HSCT) as a treatment for haematological malignancies is increasing as the benefits of this intervention are applied to a wider patient population [1]. However, although this intervention is associated with a variety of pulmonary complications, including infection and graft-versus-host disease [2,3], most of the available data has utilised regular lung function measures that are taken at least 6–12 months after the allograft [4] or retrospective clinical studies [5,6]. These do not give a detailed picture of the early effects on the lung after receiving an allograft and in particular do not quantify pulmonary inflammation during this time period. This is important as 5-45% of deaths from allogeneic HSCT (depending on indication) occur within 100 days after receiving a HSCT [1] and the cause of death can often be as a result of pulmonary complications [7]. Hence, intensive monitoring of lung physiological outcome measures may provide insights into the natural history of the impact of receiving an allogeneic HSCT on the lungs, as well as assessing if changes in respiratory function tests have prognostic value. To be applicable in the clinical setting such tests will need to be ideally readily deliverable at regular clinic visits. We thus chose spirometry (Forced Expiratory Volume in one second and Forced Vital Capacity) and exhaled nitric oxide, a measure of pulmonary inflammation, as simple measures of lung physiological health.

We have established a cohort of patients who received an allogeneic HSCT and prospectively collected data on lung function and exhaled nitric oxide to assess the impact of this intervention on pulmonary function and a biomarker of pulmonary inflammation. We have also assessed if these outcomes are associated with survival, and thus may provide a potential biomarker that could be used to stratify patients who have received a transplant.

## Methods

### Study population

Our study population consisted of 68 patients that had received an allogeneic haematological stem cell transplant (HSCT) in the Nottingham Regional Transplant Centre between January 2007 and December 2009 as standard treatment for their haematological disease. All adult patients who received an allogeneic stem cell transplant when staff were available for recruitment and who had post-transplant follow-up in Nottingham were eligible to participate in the study. The study was approved by the Nottingham Regional Ethics Committee, and written consent was obtained from all subjects. All myeloablative transplants received total body irradiation of 12 to 14.4 Gy and cyclophosphamide 60mg/kg x 2days while reduced intensity conditioning varied according to the clinical condition.

### Data collection

Approximately 1–2 weeks before the transplant, baseline data were collected on sex, age, indication for transplant and smoking history. The baseline data were collected after chemotherapy treatment had started, but prior to transplant. After transplant, the patients were assessed during their routine clinical visits to the transplant clinic at 6 weeks, 3 months and 6 months.

Pulmonary Function measurements were performed using a Vitalograph 2120 spirometer (Vitalograph Ltd, Buckingham, England). Forced vital capacity in one second (FEV_1_) and Forced Vital Capacity (FVC), were measured according to ERS/ATS criteria [8], and compared with predicted values from the European Community for Steel and Coal normal range [9]. Fractional exhaled nitric oxide (FE_NO_) measurements were made using an NIOX MINO portable analyser (Aerocrine AB, Stockholm, Sweden), an electrochemical analyser, at a sample rate of 50 mL/min. FE_NO_ measurements were performed prior to spirometry. Patients were instructed not to eat or drink for a minimum of one hour prior to testing. Patients were seated at rest, for at least 20 min prior to the test and results were recorded in parts per billion (PPB). The initial study proposal aimed to look at the short-term changes in lung function after receiving an allograft HSCT, and we were subsequently also able to later collect data on pulmonary inflammation after starting data collection.

### Statistical methods

The data were analysed using Cox Regression to assess the association of the lung measurements with subsequent survival. As well as presentation of mean changes in lung function and exhaled nitric oxide as measured by paired t-tests, the proportion of individuals whose lung function decreased by more than 15% or whose exhaled nitric oxide increased by 10ppb or more are presented, to give a more complete presentation of those who experienced large changes in the pulmonary measures of interest. The start time for the survival analysis was the date of the HSCT and the final date was the last date when the individual was followed up in clinic or the date of death. In the survival analysis, sex, age and smoking status were added to the model as *a priori* potential confounding factors. The proportional hazards assumption for the survival analysis was tested to ensure that these were not contravened. All statistical analyses were done utilising STATA 11.0 (Texas, USA). The numbers of patients followed up decreased with time as a consequence of mortality.

## Results

Between January 2007 and December 2008, 72 patients received an allogeneic transplant for haematological malignancy at the Nottingham Regional Transplant Centre and were eligible for the study. Of these, 68 (94%) patients agreed to participate in the study and 4 (6%) patients refused to consent to the study (Table 1).

**Table 1:** Characteristics of the allograft recipients in the study

**Total no. of individuals in cohort**	**68**
Male (%)	34 (50)
Mean age at transplant, sd	48.5 years (13.2)
**Smoking status (%)**	
Never	19 (28)
Ex-smoker	43 (63)
Current smoker	6 (9)
**Diagnosis at HSCT (%)**	
AML	33 (49)
ALL	8 (12)
Non Hodgkin’s Lymphoma	5 (7)
Hodgkin’s Lymphoma	5 (7)
Myelodysplastic syndrome	4 (6)
Chronic lymphatic leukaemia	4 (6)
Other*	9 (13)
**Donor type (%)**	
Matched unrelated donor	42 (62)
Cord blood	4 (6)
Sibling	22 (32)
**Stem Cell Source (%)**	
Peripheral blood stem cell source	54 (80)
Bone marrow	10 (16)
Umbilical cord	4 (6)
**Type of Conditioning prior to HSCT (%)**	
Myeloablative conditioning	23 (34)
Reduced intensity	45 (66)

*HSCT* Haematopoietic stem cell transplant.

*Sd* Standard deviation.

*Ppb* Parts per billion.

* other (n) = aplastic anaemia (1), chronic myelomonocytic leukaemia (1), chronic myeloid leukaemia (2), multiple myeloma (3) and mantle cell lymphomas (2).

After allogeneic HSCT, the lung function as measured by percent predicted FEV_1_ decreased at six weeks (− 5.9%, 95% confidence intervals CI: -8.9 to −2.9), three months (−4.7%, 95%CI: -8.4 to −1.0) and six months (−4.3%, 95% CI: -7.7 to −0.9) compared to baseline (Table 2). Similar results were seen for percent predicted FVC over the same time period. After receiving an allogeneic HSCT (Table 2), exhaled nitric oxide was increased at six weeks (+2.1ppb 95% CI: -1.8 to +5.9), three months (+6.0ppb, 95% CI: +0.4 to +11.5) and six months +6.4ppb (95% CI: +0.7 to +12.1). However, the baseline exhaled nitric oxide was not predictive of the change in FEV_1_ at three months or six months (data not shown).

**Table 2:** Change in lung function and pulmonary inflammation occurring after receiving an allograft haematopoietic stem cell transplant

	**Time after stem cell transplant**			
	**Baseline**	**6 weeks**	**3 months**	**6 months**
Mean percent predicted FEV_1_ (sd)	90.3 (15.8)	84.0 (18.9)	86.0 (19.6)	85.5 (19.4)
	N=68	N=64	N=57	N=47
Change in percent predicted FEV_1_ from baseline (95% CI)	-	- 5.9	−4.7	−4.3
		(−8.9 to −2.9)	(−8.4 to −1.0)	(−7.7 to −0.9)
		p<0.001	p=0.007	p=0.007
Number with decrease in predicted FEV_1_ by ≥15% (%)	-	10	10	8
		(16)	(18)	(17)
Mean percent predicted FVC (sd)	98.6 (15.7)	91.4 (18.1)	93.1 (18.6)	94.1 (17.1)
	N=68	N=61	N=56	N=47
Change in FVC from baseline (95% CI)		−6.4	−5.3	−5.6
	-	(−9.7 to −3.0)	(−9.1 to −1.5)	(−9.1 to −2.2)
		p<0.001	p=0.008	p=0.002
Number with decrease in predicted FVC by ≥15% (%)	-	15	10	11
		(25)	(18)	(23)
FEV_1_/FVC (sd)	76 (9)	76 (10)	77 (9)	76 (11)
	N=68	N=61	N=56	N=47
Median exhaled nitric oxide, ppb, (IQR)	15 (12–25)	18.5 (12–27)	19 (15–28)	20 (16–27)
	N=59*	N=54*	N=43*	N=38*
Change in exhaled nitric oxide from baseline, ppb (95% CI)		+2.1	+6.0	+6.4
	-	(−1.8 to +5.9)	(+0.4 to +11.5)	(+0.7 to +12.1)
		p=0.283	p=0.034	p=0.028
		N=48	N=40	N=36
Number with increase in exhaled NO by ≥10ppb (%)	-	10	11	13
		(21)	(27)	(36)

*FEV*_*1*_- Forced Expiratory Volume in one second.

*FVC*- Forced Vital Capacity.

*CI*- Confidence intervals.

*IQR*- Interquartile range.

*NO*= Nitric oxide.

* the numbers of patients with exhaled nitric oxide measurements are lower then those with lung function as this was added after data collection had begun. Statistical comparisons using paired t-tests.

Subgroup comparison between those who received full myloablative conditioning and those who received reduced intensity conditioning for revealed no consistent differences between the two groups for lung function or exhaled nitric oxide, although at three months those who received the reduced intensity conditioning had a smaller decrease in both FEV_1_ (+7.6%; 95%CI: +0.1 to +15.2) and FVC (+9.4; 95%CI: +1.6 to +17.1) compared to those who did not receive reduced intensity conditioning.

In the survival analysis, there were 22942 days of total analysis time at risk and 22 deaths during this period. After adjustment for sex, age and smoking status, there was an inverse association between change in exhaled NO from baseline to six weeks and risk of death (Hazard Ratio HR 0.95 per unit of exhaled NO, 95% CI: 0.90 to 1.00, p=0.06) and three months (HR 0.94 per unit of exhaled NO, 95% CI: 0.89 to 1.00, p=0.04) for those individuals who provided paired data. There was no association between risk of death and change in lung function at the same time points after adjusting for the same covariates (data not shown).

## Discussion

This is the first study to explore the early changes in lung function and pulmonary inflammation in a cohort of individuals who had undergone an allogeneic hematopoietic stem cell transplant. Our data demonstrate that lung function decreases and pulmonary inflammation increases after receiving an allogeneic HSCT. The change in exhaled nitric oxide may have potential in the stratification of patients for subsequent risk of mortality, and we consider that this is worth consideration in future cohort studies assessing potential biomarkers in this patient group.

One strength of our data is its high response rate, with 94% of eligible patients being enrolled in the cohort and only 6% of eligible patients declining to provide data. We were subsequently able to follow up the patients and collect detailed information on their changes in lung function and pulmonary inflammation, and also collect data on potential confounding factors and mortality. As our measurements were collected by a single person in a standardised manner, we are confident in that we have minimised the bias that can be introduced by multiple observers collecting data. The collection of data from a single centre which all patients who live in the surrounding centre attend ensures that the mortality data is complete.

Our study has a variety of limitations that require consideration. Having elected to collect detailed data on lung function and pulmonary inflammation, the study necessarily had to be from only one transplant centre due to staffing constraints. This has limited the number of participants that we were able to recruit and hence the power of the study to detect clinically important associations. However, this is the largest study to measure lung function and pulmonary inflammation in this patient cohort during the early months after receiving an allogeneic HSCT, and the 95% confidence intervals in our data should be useful in guiding researchers and clinicians in the future of the potential of these outcome measures in managing these patients. Unfortunately, we were unable to collect more detailed data on lung function including transfer factor due the logistical challenges of doing time-intensive measurements in a group of patients who are already debilitated from the HSCT. The generalisability of these data should be good for populations that are similar in age, initial disease and HSCT treatment regimens. As we have a relatively small study population and have tested a number of hypotheses, we are unable to exclude the possibility that some of the apparently significant associations observed may be spurious as a consequence of multiple hypothesis testing.

Previous research on the impact of receiving an allogeneic HSCT has tended to use routinely collected data at clinic visits, which is a powerful approach allowing over time large numbers of data to be collected. The largest previous study by Chien *et al.* demonstrated that new airflow obstruction (as defined as a more than 5% annual decline in FEV_1_ with the lowest post-transplant FEV_1_/FVC ratio of less than 0.8) was 26%, and associated with higher mortality rates at follow up than those with no airflow obstruction [4]. However, this important study did not look at pulmonary function tests within 6 months of receiving a HSCT, and hence is not directly comparable with our data. Savani *et al.*[10] studied patients who survived for five years or more after HSCT, and reported that chronic graft-versus-host disease (GVHD) and low FEV_1_ and diffusion capacity for carbon monoxide were associated with a decrease in pulmonary function. As this study described a survivor cohort with a long follow up period, again direct comparisons with our data are impossible. Similarly, Lund *et al.*[5] studied 43 patients who survived for five years and reported a decrease in lung function and transfer factor in the first year which returned to baseline after five years, and again these data are hence in a survivor cohort and not directly comparable to our data. A recent study looking at 33 patients, approximately one year after HSCT suggested that peripheral airways heterogeneity may be a better measure that could be more sensitive than simple lung function measurements such as FEV_1_ in detecting early lung pathology [11].

To our knowledge no other studies have looked at the impact of receiving an allogeneic HSCT on pulmonary inflammation in adults in the six months after the procedure. The increase in pulmonary inflammation observed after HSCT may be a consequence of either the conditioning or the result of an immunological or inflammatory response to the allograft, and the absence of a consistent difference between the two conditioning groups suggests that the latter explanation may be more likely, although the study was not powered to examine these differences**.** The inverse association between change in exhaled NO from baseline at six weeks and three months with subsequent mortality was unexpected and needs to be considered with caution as the numbers of patients studied were small and these relationships may have occurred by chance as we have tested a number of associations in this analysis (p=0.04 to 0.06). Nonetheless, this is an interesting observation worth consideration. Exhaled nitric oxide is a measure of pulmonary inflammation [12], and the inflammatory response in general probably evolved to help host organisms fight infection [13]. A recent study in 30 children with a mean age of 12 years suggests that higher exhaled nitric oxide is associated with pulmonary complications (predominantly infections) 28 days after receiving a HSCT [14], demonstrating that this biological response is active in the early period after receiving a HSCT. Hence, we cautiously speculate that the inverse association between change in pulmonary inflammation and mortality observed in our dataset, if a true phenomenon, may be a consequence of an impaired ability to generate an inflammatory response, which may then result in increased susceptibility to infections in this patient group.

By adding to the literature of the relatively short-term consequences of receiving an allogeneic HSCT on the lungs, we have observed associations and hence generated hypotheses that now require consideration in other populations. The identification of biomarkers for pulmonary disease and mortality in individuals who have received an allogeneic HSCT is important as it will allow the early identification of those who are at high risk for these complications. This can then permit more intensive monitoring of this patient group and possibly also stratification to allow better targeted trials of interventions that can modify disease progression without exposing individuals who are less likely to develop these complications to the potential adverse side-effects that can accompany many therapeutic interventions.

## Conclusions

In summary, we have demonstrated in a representative sample of patients who received an allogeneic HSCT that pulmonary inflammation increases in the first six months after the procedure while lung function decreases over the same time period. We hope that future research in this area will examine these associations in larger numbers of patients that will provide more power to determine the clinical importance of these associations.

## Abbreviations

FEV_1_: Forced Expiratory Volume in one second; FVC: Forced Vital Capacity; NO: Nitric oxide; HSCT: Human stem cell transplant; GVHD: Graft-versus-host disease.

## Competing interests

None of the authors have a competing of interests to declare in relation to this work.

## Authors’ contribution

Conception and design: AF, RH, JB, E D-G, NR; Data Collection: AE, LW, Analysis and interpretation: AE, TM, AF, RH; Drafting the manuscript for important intellectual content: all authors. All authors read and approved the final manuscript.

## Pre-publication history

The pre-publication history for this paper can be accessed here:

http://www.biomedcentral.com/1471-2466/13/2/prepub
